# Longitudinal Trends in Tobacco Availability, Tobacco Advertising, and Ownership Changes of Food Stores, Albany, New York, 2003–2015

**DOI:** 10.5888/pcd13.160002

**Published:** 2016-05-12

**Authors:** Akiko S. Hosler, Douglas H. Done, Isaac H. Michaels, Diana C. Guarasi, Jamie R. Kammer

**Affiliations:** Author Affiliations: Douglas H. Done, Isaac H. Michaels, Diana C. Guarasi, Jamie R. Kammer, Department of Epidemiology and Biostatistics, University at Albany School of Public Health, Rensselaer, New York.

## Abstract

**Introduction:**

Frequency of visiting convenience and corner grocery stores that sell tobacco is positively associated with the odds of ever smoking and the risk of smoking initiation among youth. We assessed 12-year trends of tobacco availability, tobacco advertising, and ownership changes in various food stores in Albany, New York.

**Methods:**

Eligible stores were identified by multiple government lists and community canvassing in 2003 (n = 107), 2009 (n = 117), 2012 (n = 135), and 2015 (n = 137). Tobacco availability (all years) and advertising (2009, 2012, and 2015) were directly measured; electronic cigarettes (e-cigarettes) were included in 2015.

**Results:**

Percentage of stores selling tobacco peaked at 83.8% in 2009 and declined to 74.5% in 2015 (*P* for trend = .11). E-cigarettes were sold by 63.7% of tobacco retailers. The largest decline in tobacco availability came from convenience stores that went out of business (n = 11), followed by pharmacies that dropped tobacco sales (n = 4). The gain of tobacco availability mostly came from new convenience stores (n = 24) and new dollar stores (n = 8). Significant declining trends (*P* < .01) were found in tobacco availability and any tobacco advertising in pharmacies and in low (<3 feet) tobacco advertising in convenience stores and stores overall. Only one-third of stores that sold tobacco in 2003 continued to sell tobacco with the same owner in 2015.

**Conclusion:**

The observed subtle declines in tobacco availability and advertising were explained in part by local tobacco control efforts, the pharmacy industry’s self-regulation of tobacco sales, and an increase in the state’s tobacco retailer registration fee. Nonetheless, overall tobacco availability remained high (>16 retailers per 10,000 population) in this community. The high store ownership turnover rate suggests that a moratorium of new tobacco retailer registrations would be an integral part of a multi-prong policy strategy to reduce tobacco availability and advertising.

## Introduction

Most tobacco retailers also sell food (eg, convenience stores, supermarkets, and drug stores) and are the primary community locations where people of all ages are exposed to tobacco products and pro-tobacco messages. Studies of youth tobacco-related behavior indicate that frequency of visiting convenience and corner grocery stores that sell tobacco is positively associated with the odds of ever smoking and the risk of smoking initiation (1,2). Largely unregulated point-of-sale tobacco advertising entices experimental smoking by adolescents and encourages experimental smokers to become regular smokers (3,4). Point-of-sale tobacco advertising is associated positively with illegal tobacco purchases by underaged youth (5). At the community level, both youth and adult smoking is positively associated with densities of tobacco retailers in the neighborhood where they live (6–9). Furthermore, proximity to tobacco retail outlets triggers stronger urges to smoke (10) and reduces the likelihood of smoking cessation by adult smokers (11).

As part of a comprehensive tobacco control strategy, the public health community has experimented with promoting laws against tobacco sales in pharmacies and grocery stores and regulations that curtail the number or density of tobacco outlets in communities and near schools (12–15). Determining the feasibility of such approaches requires an understanding of the tobacco retail environment. However, research findings on the tobacco environment are mostly cross-sectional. Studies that examined long-term changes in tobacco availability and advertising are scarce. 

The purpose of this study is to describe and explain trends in directly measured tobacco availability, tobacco advertising, and ownership changes in various types of retail stores selling food in a defined urban community from 2003 to 2015. We also assessed the availability and advertising of electronic cigarettes (e-cigarettes) in 2015, because this new product is now the most used tobacco product among middle- and high-school students (16).

## Methods

The setting of this study was 6 zip code areas in downtown Albany, New York. This area has been designated as a priority community of our university’s research center since 2002 because of elevated chronic disease risks of its residents, including high prevalence of smoking (17). On the basis of decennial population census data, the study area had a population of 52,700 in 2000 and 54,100 in 2010, or about 55% of the city’s total population in both years. Approximately 42% of the area residents were African-American, 8% were Hispanic, and 4% were Asian, with the poverty rate of 34%.

Data collection took place from June through August in 2003, 2009, 2012, and 2015. We defined an eligible store as a retail outlet that sold at least one of the following indicator food items: fresh milk, bread, or fruits or vegetables that were fresh, frozen, or canned. Stores that were located inside the access-restricted area of an office building were excluded. We initially identified locations of stores by combining various administrative lists of retailers obtained from government agencies (ie, lists of inspected food retailers, registered tobacco retailers, off-premises liquor license holders, Supplemental Nutrition Assistance Program authorized retailers, and authorized lottery retailers). Our team of trained survey takers systematically canvassed the study area to verify the eligibility of all listed stores and find additional eligible stores not on the lists. With permission from the store owner or manager, we conducted an in-store observational assessment using a paper tool called the Food Retail Outlet Survey Tool. This tool had excellent interrater agreement of all tobacco measures (κ ≥ 0.90) (18). All eligible stores granted permission to conduct an in-store assessment in all data collection years. The University at Albany institutional review board reviewed and approved the study protocols.

Tobacco availability was measured by the presence of any tobacco products for sale in 2003, 2009, 2012, and 2015. We used the registered tobacco retailers’ lists obtained from the New York State Department of Taxation and Finance to verify the legality of selling tobacco and to collect store ownership information. We also used the New York State Department of Health’s Youth Access Tobacco Enforcement Annual Reports to identify any disciplinary actions on retailers that resulted in tobacco registration suspension or revocation. In 2009, 2012, and 2015, indoor tobacco advertising was measured by the presence of any objects bearing the name, image, or both of a tobacco brand that were placed inside the store. The platforms of advertising included stickers, posters, plaques, price cards, and banners, as well as free-standing pieces such as counter mats and change trays. Tobacco advertising placed at the eye level of a young child (<3 feet) was measured separately and identified in this study as “low tobacco advertising.” Availability of e-cigarettes and indoor advertising for e-cigarettes were measured in 2015 only.

Data on supermarkets, convenience stores (with or without gas pumps), pharmacies, dollar stores, and specialty food stores (ie, stores that sell food products such as produce, meat, fish, dairy, baked goods, ethnic groceries, or natural foods) were retained for this study. We excluded seasonal outdoor markets and produce trucks.

Counts and percentages of stores selling tobacco or e-cigarettes and having their advertising inside the store were tabulated for each relevant data collection year. We used the χ^2^ trend test to evaluate significance of the changes in percentages over time. Reasons for changes of tobacco availability, whether it was because of tobacco sale status change (dropped or added tobacco sales) or business conversion (closing or opening of stores) were examined by tracking stores longitudinally. We defined “existing store” as a store that has been operating the same type of food business at the same address, regardless of a change in appearance, name, or ownership. A store that “went out of business” was defined as a store that disappeared physically (boarded up or demolished) or was transformed into a different type of food or nonfood business. All analyses were repeated for each type of store. We also tracked changes in ownership for a baseline cohort of stores that sold tobacco in 2003. SPSS version 23.0 (IBM Corporation) was used for data analysis. In addition to quantitative analysis, we conducted an informal interview with 2 key members of the tobacco-free coalition based in Albany in 2015 and collected information about their activities with regard to reducing tobacco availability and advertising in stores.

## Results

Eligible stores included for this study were 107 in 2003, 117 in 2009, 135 in 2012, and 137 in 2015.

### Tobacco availability and advertising

The number of stores selling tobacco peaked at 107 in 2012 and declined slightly to 102 in 2015 (Table 1). Percentage of stores selling tobacco peaked at 83.8% in 2009 and gradually declined to 74.5% in 2015, but the trend was not significant (*P* = .11). Convenience stores and supermarkets continued to have high percentages (80%–100%) of stores selling tobacco, and dollar stores had increasing percentages of stores selling tobacco (*P* = .08). Pharmacies were the only type of store in which percentage of stores selling tobacco significantly declined, from 100% in 2003 and 2009 to 50% in 2015 (*P* = .003). All stores selling tobacco were registered properly to sell tobacco.

**Table 1:** Proportions of Stores Selling Tobacco and E-Cigarettes, and Having Tobacco and E-Cigarette Advertising, by type of Store, Albany, New York, 2003–2015^a^

Characteristic	Supermarket	Convenience Store	Pharmacy	Dollar Store	Specialty Food Store	Total
	n/N (%)					
**Sold tobacco**						
2003	3/3 (100)	72/73 (98.6)	8/8 (100)	0/2 (0)	4/21 (19.0)	87/107 (81.3)
2009	3/3 (100)	78/78 (100)	9/9 (100)	3/5 (60.0)	5/22 (22.7)	98/117 (83.8)
2012	4/5 (80.0)	82/83 (98.8)	8/9 (88.9)	6/9 (66.7)	7/29 (24.1)	107/135 (79.3)
2015	4/5 (80.0)	83/85 (97.6)	5/10 (50.0)	7/9 (77.8)	3/28 (10.7)	102/137 (74.5)
*P* for trend	.30	.43	.003	.08	.46	.11
**Tobacco advertisement**						
2009	0/3 (0)	74/78 (94.9)	9/9 (100)	3/5 (60.0)	3/22 (13.6)	89/117 (76.1)
2012	0/5 (0)	78/83 (94.0)	8/9 (88.9)	6/9 (66.7)	3/29 (10.3)	95/135 (70.4)
2015	0/5 (0)	76/85 (89.4)	5/10 (50.0)	7/9 (77.8)	1/28 (3.6)	89/137 (65.0)
*P* for trend	NA	.17	.007	.47	.21	.05
**Low tobacco advertisement**						
2009	0/3 (0)	45/78 (57.7)	0/9 (0)	0/5 (0)	1/22 (4.5)	46/117 (39.3)
2012	0/5 (0)	25/83 (30.1)	0/9 (0)	0/9 (0)	0/29 (0)	25/135 (18.5)
2015	0/5 (0)	16/85 (18.8)	0/10 (0)	0/9 (0)	0/28 (0)	16/137 (11.7)
*P* for trend	NA	<.001	NA	NA	.17	<.001
**Sold e-cigarettes**						
2015	0/5 (0)	54/85 (63.5)	5/10 (50.0)	5/9 (55.6)	1/28 (3.6)	65/137 (47.4)
**E-cigarette advertisement**						
2015	0/5 (0)	52/85 (61.2)	0/10 (0)	5/9 (55.6)	0/28 (0)	57/137 (41.6)
**Low e-cigarette advertisement**						
2015	0/5 (0)	2/85 (2.4)	0/10 (0)	0/9 (0)	0/28 (0)	2/137 (1.5)

Abbreviation: NA, not applicable.

a “n” represents the number of stores with a specific characteristic; “N” represents the total number of stores.

Indoor tobacco advertising was found in 76.1% of all stores in 2009, then gradually declined to 65.0% in 2015, although the trend was not significant (*P* = .05). Common platforms of advertising were hanging cigarette price cards and plates attached to tobacco manufacturer-supplied cigarette display cases. Most convenience stores had advertising, while no supermarkets had advertising in all 3 years. Additionally, all supermarkets eliminated tobacco display shelves and made tobacco products totally invisible from customers before the 2009 data collection. We learned that this was the result of a targeted campaign by members of the local tobacco-free coalition, who successfully negotiated with all major regional supermarket chains to cover up tobacco products in 2007 and 2008. Pharmacies were the only type of stores with a significant decline of having any indoor tobacco advertising, from 100% in 2009 to 50% in 2015 (*P* = .007).

Convenience stores were most likely to have low tobacco advertising (57.7% in 2009). However, the proportion of convenience stores having low tobacco advertising declined significantly to 18.8% in 2015 (*P* < .001). Supermarkets, pharmacies, and dollar stores had no low tobacco advertising at all in all data collection years. Stores overall also had a significant decline in low advertising, from 39.3% (46 stores) in 2009 to 11.7% (16 stores) in 2015 (*P* < .001).

### E-cigarette availability and advertising

E-cigarettes were sold in 47.4% of all stores, or 63.7% of stores selling tobacco in 2015. Convenience stores were most likely to sell e-cigarettes (63.5%), followed by dollar stores (55.6%) and pharmacies (50.0%). No supermarkets sold e-cigarettes, and all e-cigarette-selling stores were registered to sell tobacco.

All dollar stores that sold e-cigarettes (5 of 5) and nearly all convenience stores that sold e-cigarettes (52 of 54) had indoor advertising of the product; supermarkets, pharmacies, and specialty stores had no advertising for e-cigarettes. Some pharmacies strategically placed e-cigarettes next to tobacco cessation products. Common platforms of e-cigarette advertising were manufacturer-supplied display cases and brand-name stickers. Low advertising of e-cigarettes was rare and found only in 2 convenience stores.

### Reasons for tobacco availability change

The most common reason for the decrease in tobacco availability was stores that sold tobacco going out of business (Table 2). Sixteen such stores, of which 11 were convenience stores, went out of business during the 12-year study period. Conversely, the most common reason for the increase in tobacco availability was opening new stores that sold tobacco. A total of 39 new stores selling tobacco were added in this community in the 12-year period, with most of them being convenience stores (n = 24) or dollar stores (n = 8). 

**Table 2:** Changes in Tobacco Availability in Food Stores, by Number of Stores, Albany, New York, 2003–2015

Characteristic^a^	Supermarket	Convenience Store	Pharmacy	Dollar Store	Specialty Food Store	Total
**Stopped selling tobacco**						
2003–2009	0	0	0	0	0	0
2009–2012	0	0	−1	0	−1	−2
2012–2015	0	−2	−3	0	−2	−7
Total	0	−2	−4	0	−3	−9
**Went out of business**						
2003–2009	0	−2	−1	0	0	−3
2009–2012	0	−3	0	−1	0	−4
2012–2015	0	−6	0	−1	−2	−9
Total	0	−11	−1	−2	−2	−16
**Started selling tobacco**						
2003–2009	0	0	0	0	0	0
2009–2012	0	0	0	0	0	0
2012–2015	0	0	0	1	0	1
Total	0	0	0	1	0	1
**Newly opened**						
2003–2009	0	8	2	3	1	14
2009–2012	1	7	0	4	3	15
2012–2015	0	9	0	1	0	10
Total	1	24	2	8	4	39
**Net change**						
2003–2009	0	6	1	3	1	11
2009–2012	1	4	−1	3	2	9
2012–2015	0	1	−3	1	−4	−5
Total	1	11	−3	7	−1	15

a The assessment period began and ended during the summer of each year.

A change of tobacco sale status in existing stores was much less common. Only one existing store (a dollar store) added the sale of tobacco during the entire study period. Nine stores, (4 pharmacies, 3 specialty food stores, and 2 convenience stores) dropped tobacco sales, and most of them (n = 7) did so during the 2012 to 2015 period. None of these stores had a record of tobacco registration suspension or revocation, indicating that the drop of tobacco sales was voluntary.

Among the 4 pharmacies that dropped tobacco sales, 3 were CVS pharmacy chain stores, which became tobacco-free according to company policy in September 2014 (19), and the remaining pharmacy was independently owned. We learned that the local tobacco-free coalition was directly responsible for the drop of tobacco sales in the independent pharmacy. Members of the coalition repeatedly met with pharmacy owners in the region and persuaded them to discontinue selling tobacco in 2011.

### Ownership change

Table 3 presents a summary of ownership changes for the baseline (2003) cohort of 87 stores selling tobacco. Seventy-three stores (83.9%) continuously sold tobacco, but only 29 of them (33.3%) had the same owner for the 12-year period. Two stores had as many as 4 owners in the same period. An additional analysis found that 19 of the cohort of 87 stores were corporate-owned pharmacies, supermarkets, and convenience stores. Although 68.4% of corporate-owned stores continuously sold tobacco without ownership changes, only 23.5% of independently owned stores did the same. This difference was significant at *P* < .01 (data not shown).

**Table 3:** Business Status and History of Ownership Transfers, Baseline Cohort of 87 Stores Selling Tobacco, by Number of Stores, Albany, New York, 2003–2015

Characteristic	1 Owner	2 Owners	3 Owners	4 Owners	Total
Sold tobacco continuously	29	29	13	2	73
Stopped selling tobacco	5	1	0	0	6
Went out of business	4	2	2	0	8
Total	38	32	15	2	87

## Discussion

To the best of our knowledge, this study is the first to examine long-term trends of directly measured tobacco availability, tobacco advertising, and ownership changes in retail stores selling food in a defined community. The longitudinal design allowed us to investigate how and why tobacco availability and advertising changed over time.

During the study period, New York State enacted legislation aimed at reducing tobacco users, including expanding the comprehensive New York State Clean Indoor Air Act in 2003, substantial increases in the state tobacco excise tax in 2008 (from $1.50 to $2.75 per pack) and again in 2010 (to $4.38 per pack), and several amendments of the Youth Access Tobacco Control Law. In 2011, the annual tobacco retailer registration fee was also raised from $100 to $300 (20,21). Despite this anti-tobacco legislation, tobacco remained widely available in this study community. Estimated per 10,000 population, tobacco retailer densities were 16.4 in 2003, 18.2 in 2009, 19.7 in 2012, and 18.7 in 2015. These density figures fell in the highest range reported by a study conducted in comparable midsize cities in California (8). A further analysis indicated that the number of tobacco-selling stores increased at a greater pace in minority neighborhoods (census block groups with racial/ethnic minorities ≥50%) compared with nonminority neighborhoods. (Figure). We hypothesized that this was explained in part by a higher smoking rate in minority populations, as well as greater availability of affordable commercial properties suited for tobacco retail businesses in the minority neighborhoods. Albany County (where the study community is located) had only a 2% decline in adult smoking prevalence from 2003 to 2009, whereas adjacent urban counties had a nearly 25% decline during the same period (22).

**Figure:**
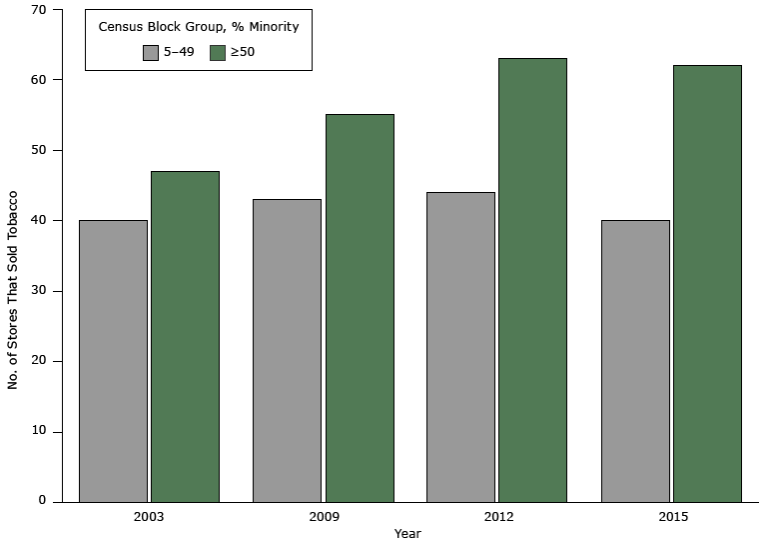
Number of Stores Selling Tobacco, by Racial/Ethnic Composition of Census Block Groups, Albany, New York, 2003–2015. **Table:** 

Year	Census Block Group, % Racial/Ethnic Minority	
	5–49	≥50
2003	40	47
2009	43	55
2012	44	63
2015	40	62

A small number of stores voluntarily dropped tobacco sales: 9 from 2009 through 2015, of which 5 were independently owned convenience stores or specialty food stores. These 5 stores had only 1 cash register each, and their average business hours (76 hours per week) were significantly (*P* < .01) shorter than those of other stores selling tobacco (113 hours per week), indicating that they were low-volume retailers (data not shown). Voluntary withdrawal of tobacco from convenience stores is rare, and most store owners are not willing to give up selling tobacco products even when incentives are provided (23). We hypothesized that the increase in tobacco registration fee in 2011 was most likely responsible for the drop of tobacco sales in these low-volume retailers. Dropping tobacco sales by chain and independent pharmacies also contributed to a small decline in tobacco availability. This fact reflected a growing social pressure toward a ban on tobacco sales in pharmacies by health care communities (24,25), consumers (12), and policy makers (13), as well as the successful targeted campaign by the local tobacco-free coalition.

For tobacco advertising, we observed that the in-store environment has become less pro-tobacco. The significant decline in low tobacco advertising in all stores — and convenience stores in particular — were important milestones, because the likelihood of young children being exposed to pro-tobacco messages in stores was reduced. Tobacco advertising attached to product display (ie, display cases and price cards), however, was still common in most stores, except in supermarkets and specialty food stores. Pharmacies that were not tobacco-free continued to have open tobacco displays and indoor advertising. A 2011 study of retail tobacco advertising in New York State reported that pharmacies had a 56% bigger space for tobacco display than did all other tobacco retailers. The researchers recommended that pharmacies should be targeted for further campaigns to eliminate point-of-sale advertising (26).

E-cigarettes were available in nearly two-thirds of stores selling tobacco (63.7%). This figure was higher than the previously reported 31% to 34% in a sample of tobacco retailers in the United States (27), 53% in licensed tobacco retailers in Kentucky counties (28), and 59.9% in tobacco retailers in 11 college communities in North Carolina and Virginia (29), suggesting that e-cigarettes are becoming increasingly more available in retail stores. Literature confirms that the market for e-cigarettes has grown rapidly, resulting in a 321% increase in sales during 2012 and 2013 in the United States (30). Nonetheless, e-cigarettes represent only approximately 1% of total tobacco product sales in the United States (30). 

Finally, we found that stores selling tobacco had a high business turnover rate; only one-third of the tobacco-selling stores identified at baseline continued to sell tobacco with the same owner in 2015. In New York State, all new store owners must file a tobacco registration at the Department of Taxation and Finance to legally sell tobacco. This requirement creates an opportunity for state and local governments to regulate tobacco availability by setting limits on the number, location, or types of retailers (20). For this community to have a moderate density (<14.0 per 10,000) of tobacco retailers (8), approximately 26 retailers should be eliminated from the 2015 count of 102. We estimated that such a reduction could be achieved if no new registrations were granted for 3 to 4 years. Prohibiting sales of tobacco in pharmacies (including supermarkets with a pharmacy department) would reduce 9 tobacco retailers. Because mostly corporate-owned pharmacies do not change ownership as often as independent convenience stores do, an additional policy approach targeting the elimination of tobacco sales in pharmacies is viable.

Our study has limitations. We focused on stores selling food, because they are the most common and influential type of retailer for community tobacco exposure; however, there are other types of tobacco or e-cigarette retailers from whom we did not collect information. For each data collection year, fewer than 10 bars, clubs, and smoke shops that registered to sell tobacco existed in this community. Additionally, an estimated 3 to 5 “vape shops” that sold e-cigarettes without New York State tobacco retailer registrations existed. Intervals between data collections were 3 to 6 years, which were sufficiently long to observe significant changes, but the intervals were not sensitive enough to identify a specific point when a change had occurred. We did not collect information on the availability and advertising of tobacco cessation products and medications. The conclusions derived from this study may not be generalizable beyond this study community.

Tobacco availability and advertising in stores can change subtly in response to tobacco control efforts by local public health advocates and an increase in the state tobacco retailer registration fee. The pharmacy industry’s self-regulation on the sale of tobacco can also contribute to changes. However, these changes are small and not sufficient to significantly improve the retail tobacco environment overall. Our study community experienced a net increase of 15 tobacco retailers in the 12-year period. Persistent tobacco advertising and a wide availability of e-cigarettes in convenience stores and dollar stores were also noted. Because stores selling tobacco have a high ownership turnover rate, a legislation to enforce a moratorium on new tobacco registrations may be a feasible approach to curb the excess availability of tobacco in this community. An additional policy approach to eliminate tobacco sales in pharmacies and continuous support for local tobacco control efforts targeting minority neighborhoods may be feasible.
